# Assessment of microvascular flow by Doppler in prostate biopsy: correlation with the Gleason score

**DOI:** 10.1590/0100-3984.2025.0070

**Published:** 2025-12-08

**Authors:** João Vitor de Oliveira, Alexandre Peroni Borges, Rodrigo Menezes Jales, Konrado Tenorio, Eduardo Miquelino de Oliveira Junior

**Affiliations:** 1 Universidade Estadual de Campinas (Unicamp), Campinas, SP, Brazil; 2 Centro Médico de Campinas, Campinas, SP, Brazil

**Keywords:** Prostatic neoplasms, Ultrasonography, Doppler, Neoplasm grading, Biopsy, needle, Neoplasms/diagnosis., Neoplasias da próstata, Ultrassonografia Doppler, Gradação de tumores, Bópsia por agulha, Neoplasias/diagnóstico.

## Abstract

**Objective:**

To determine whether the degree of microvascular flow on Doppler ultrasound correlates with the aggressiveness of prostate cancer, as defined by the Gleason score.

**Materials and Methods:**

This was a prospective cohort study including 88 patients evaluated between November 2023 and July 2024. We included men between 48 and 85 years of age with a prostate-specific antigen (PSA) level between 0.3 ng/mL and 21.0 ng/mL and an imaging finding with Prostate Imaging-Reporting and Data System (PI-RADS) category between 2 and 5. Patients with indeterminate biopsy results were excluded, as were those for whom PSA values were missing, those who did not undergo microvascular Doppler assessment, and those previously diagnosed with prostate cancer. In each case, we performed systematic 10-core transrectal biopsy, guided by 1.5-T magnetic resonance imaging-ultrasound fusion, as well as performing Doppler ultrasound with microvascular flow imaging. Vascularization was qualitatively assessed and categorized as absent/minimal, moderate, or marked. The Gleason score was classified as clinically significant (≥ 7) or not (≤ 6)..

**Results:**

A significant association was found between the degree of microvascular flow and the Gleason score (*p* = 0.0384). Spearman’s correlation was moderate (r = 0.377), and Kendall’s tau was 0.300, indicating a positive relationship between higher microvascular flow and greater tumor aggressiveness.

**Conclusion:**

Microvascular Doppler shows potential as a complementary tool in prostate biopsy, enabling more precise targeting of regions with increased vascularity, which might be associated with greater tumor aggressiveness.

## Abstract (pt)

**Objetivo:**

Avaliar se há correlação entre o grau de fluxo microvascular ao Doppler e a agressividade do câncer de próstata definida pelo escore de Gleason.

**Materiais e Métodos:**

Estudo de coorte prospectivo com 88 pacientes entre novembro de 2023 e julho de 2024. Foram incluídos homens entre 48 e 85 anos, com PSA entre 0,3 e 21 ng/mL e laudos PI-RADS 2 a 5. Excluíram-se pacientes com bópsia indeterminada, PSA ausente, sem Doppler microvascular ou com diagnóstico prévio de câncer de próstata. Realizou-se bópsia transretal sistemática com 10 fragmentos, guiada por fusão com ressonância magnética (1,5-T) e ultrassonografia com Doppler de fluxo microvascular. A vascularização foi avaliada qualitativamente e o fluxo foi categorizado como ausente/mínimo, moderado ou acentuado. O escore de Gleason foi classificado como clinicamente significativo (≥ 7) ou não significativo (≤ 6).

**Resultados:**

Houve associação significativa entre o grau de fluxo microvascular e o escore de Gleason (*p* = 0,0384). A correlação de Spearman foi moderada (r = 0,377) e o tau de Kendall foi de 0,300, indicando relação positiva entre maior grau de fluxo e maior agressividade tumoral.

**Conclusão:**

O Doppler microvascular demonstrou potencial como ferramenta complementar à bópsia prostática, permitindo direcionamento mais preciso para regiões com maior vascularização, possivelmente associadas a tumores mais agressivos.

## INTRODUCTION

Prostate cancer (PCa) became the second leading cause of cancer-related death among adult men worldwide in 2022^**(^1^)**^. In Brazil, it was responsible for 16,429 deaths that same year^**(^2^)**^, whereas 31,620 PCa-related deaths were recorded in the United States in 2019^**(^3^)**^.

Currently, prostate-specific antigen (PSA) testing and digital rectal examination are recommended for PCa screening. Magnetic resonance imaging (MRI) techniques are also employed to differentiate cancerous from non-cancerous tissues, improving diagnostic accuracy^**(^4^,^5^)**^.

Transrectal ultrasound-guided biopsy remains a cost-effective and widely available diagnostic technique. New ultrasonographic techniques, such as microvascular flow (MVF) imaging, enable detailed visualization of blood flow in small vessels-particularly in areas around and including tumors, which tend to be more vascularized^**(^6^)**^. Evidence suggests that such vascular patterns are also associated with tumor aggressiveness in renal, hepatic, and cervical cancer^**(^7^-^9^)**^.

The objective of this study was to determine whether the degree of MVF on Doppler ultrasound correlates with the aggressiveness of PCa, as defined by the Gleason score.

## MATERIALS AND METHODS

This was a prospective cohort study with non-randomized exposure categorization, conducted in the city of Campinas, Brazil, from November 2023 to July 2024. A total of 88 patients were eligible for inclusion. Patients were included if they me the following criteria: being a male; being 48-85 years of age; having a PSA level between 0.3 ng/mL and 21.0 ng/mL; and having an imaging finding assigned a Prostate Imaging-Reporting and Data System (PI-RADS) category between 2 and 5. Patients with indeterminate biopsy results were excluded, as were those for whom PSA values were missing, those who did not undergo microvascular Doppler assessment, and those previously diagnosed with PCa. Thus, 21 patients were excluded. Therefore, the final sample comprised 67 patients.

### Procedure

**Figure 1:**
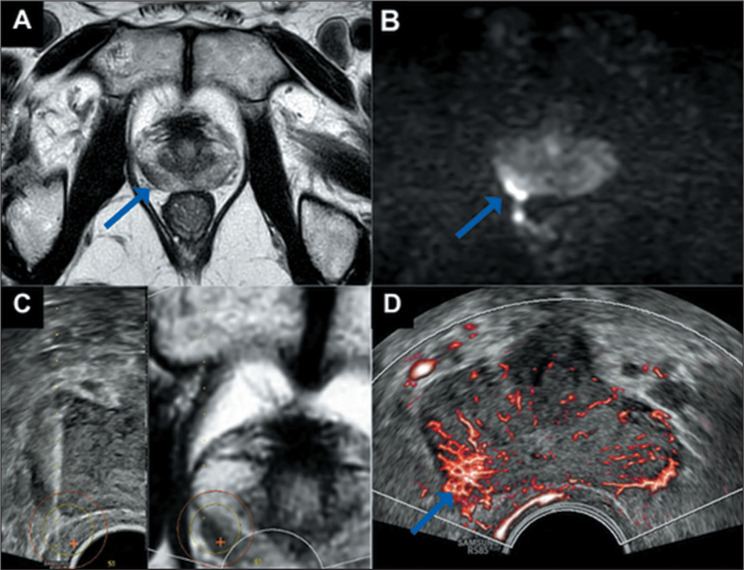
Example of the assessment. A 74-year-old patient with a PSA level of 3.09 ng/mL. A: Nodular area with low signal intensity on T2-weighted imaging in the transition zone and peripheral zone (14 × 8 mm). B: Restricted diffusion on diffusion-weighted imaging at a b-value of 2,000 s/mm^2^, with corresponding hypointensity on the apparent diffusion coefficient map. C: Fusion of the suspicious MRI area (PI-RADS category 4) with the ultrasound image. D: Macrovascular Doppler technique demonstrating increased MVF in the suspicious area.

Patients were placed in the left lateral position under anesthesia. Systematic biopsy was performed with 10 cores using an 18-G tru-cut needle via the transrectal route. The MRI scans (minimum 1.5-T) were fused with real-time ultrasound images acquired by using a high-resolution ultrasound system (RS85 Prestige; Samsung Medison Co Ltd, Seoul, South Korea). Microvascular Doppler ultrasound was applied to the MRI-identified suspicious area (Figure 1). All procedures were performed by an operator with over 15 years of experience.

### Image assessment

Image analysis was conducted by a radiologist with more than 10 years of experience who did not participate in the procedure and was blinded to the histopathological results. Qualitative assessment of vascularization patterns was based on dynamic images recorded during the biopsy.

### Categorization

The Gleason score is a histopathological score that assesses tumor aggressiveness on the basis of biopsy samples^**(^10^)**^. Patients were divided into two groups, by Gleason score (Table 1): ≥ 7 (clinically significant; n = 30); and ≤ 6 (clinically insignificant; n = 37).

**Table 1:** Distribution of patients with PCa, by Gleason score.

Gleason score	(N = 67)
0, n (%)	26 (38.8)
6, n (%)	11 (16.4)
7, n (%)	26 (31.3)
8, n (%)	1 (1.5)
9, n (%)	5 (7.5)
10, n (%)	3 (4.5)

### MVF grading

**Figure 2:**
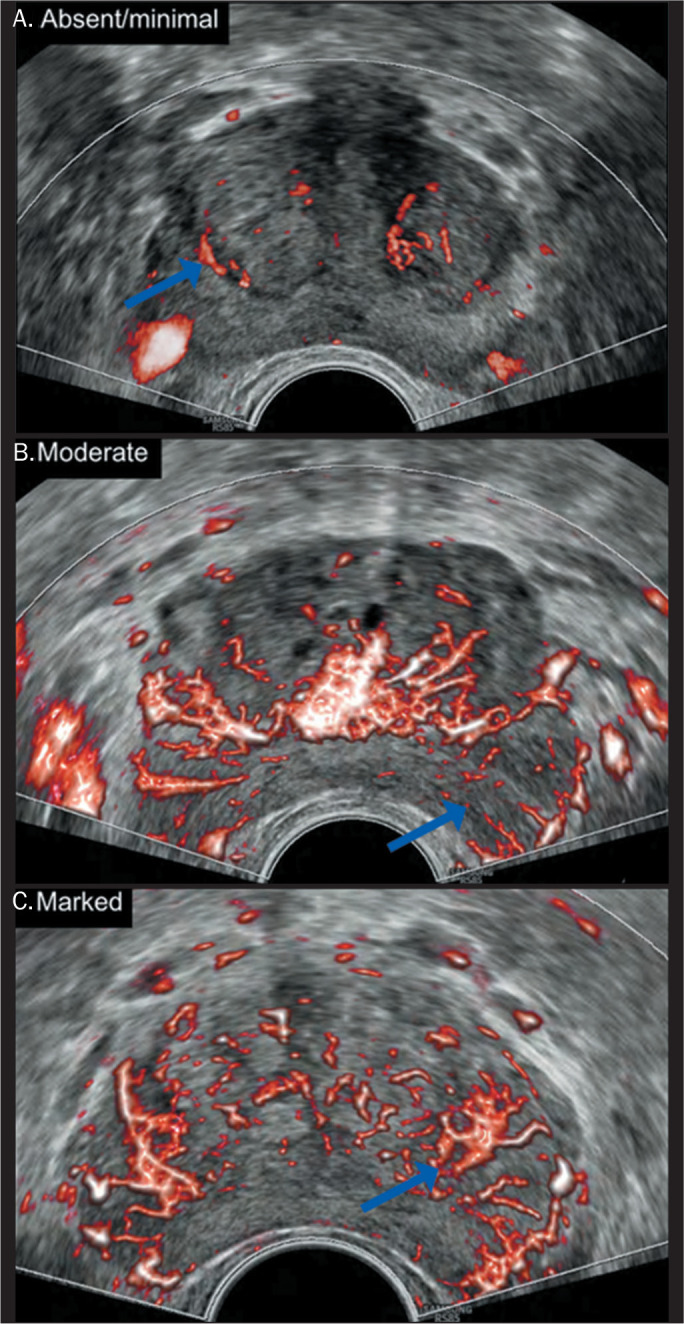
Reference criteria for flow analysis in suspicious areas identified on MRI (arrows). A: Absent or minimal flow compared with the pattern seen in unsuspicious areas. B: Moderate flow compared with the pattern seen in unsuspicious areas. C: Marked flow compared with the pattern seen in unsuspicious areas.

Vascular flow was categorized on the basis of the MVF pattern in the MRI-identified suspicious area (Figure 2), in comparison with the rest of the gland, as follows: absent or minimal; moderate; or marked.

### Statistical analysis

The primary analysis evaluated the association between MVF grade and tumor aggressiveness (Gleason score). The chi-square test was used in order to assess associations between categorical variables. Spearman’s rank correlation (ρ) and Kendall’s tau (τ) were used as ordinal measures of monotonic association. Values of *p* < 0.05 were considered statistically significant. Data analysis was performed with the IBM SPSS Statistics software package, version 27.0 (IBM Corp., Armonk, NY, USA). Table 2 summarizes the PSA levels, PI-RADS categories, clinically significant biopsy status, and highest Gleason scores.

**Table 2:** Patient-level data.

Patient ID	PSA level (ng/mL)	PI-RADS category	Clinically significant biopsy status	Highest Gleason score
1	5.44	5	Yes	10
2	9.98	5	Yes	10
3	13.0	5	Yes	10
4	6.33	4	Yes	9
5	6.99	5	Yes	9
6	8.18	4	Yes	9
7	11.22	5	Yes	9
8	12.37	4	Yes	9
9	3.5	5	Yes	8
10	2.41	4	Yes	7
11	2.72	5	Yes	7
12	3.09	4	Yes	7
13	3.19	5	Yes	7
14	3.4	4	Yes	7
15	4.1	4	Yes	7
16	4.41	4	Yes	7
17	5.17	4	Yes	7
18	5.2	5	Yes	7
19	5.35	2	Yes	7
20	5.4	5	Yes	7
21	5.98	5	Yes	7
22	6.15	4	Yes	7
23	6.52	4	Yes	7
24	6.77	4	Yes	7
25	7.4	4	Yes	7
26	7.87	5	Yes	7
27	8.97	5	Yes	7
28	12.38	5	Yes	7
29	13.0	5	Yes	7
30	19.4	4	Yes	7
31	0.3	4	No	6
32	2.32	4	No	6
33	3.0	4	No	6
34	3.01	4	No	6
35	3.57	4	No	6
36	4.65	4	No	6
37	5.48	4	No	6
38	9.19	4	No	6
39	9.62	4	No	6
40	10.85	4	No	6
41	21.0	4	No	6
42	0.34	3	No	0
43	1.97	4	No	0
44	3.0	3	No	0
45	3.43	4	No	0
46	3.64	4	No	0
47	4.17	4	No	0
48	4.2	4	No	0
49	4.65	3	No	0
50	4.68	4	No	0
51	4.8	5	No	0
52	5.2	4	No	0
53	5.21	4	No	0
54	5.66	4	No	0
55	6.28	4	No	0
56	7.49	4	No	0
57	7.5	4	No	0
58	8.05	4	No	0
59	8.4	4	No	0
60	8.89	4	No	0
61	9.1	4	No	0
62	9.7	5	No	0
63	9.72	4	No	0
64	12.83	2	No	0
65	12.9	4	No	0
66	8.66	3	No	0
67	11.37	5	No	0

## RESULTS

A statistically significant association was observed between MVF grade and tumor aggressiveness, as quantified by the Gleason score (*p* = 0.0384). We detected a moderate positive correlation between the MVF grade and Gleason score (ρ = 0.377), and we identified a consistent monotonic relationship between the two (τ = 0.300). We present the comprehensive patient data in Table 2 and a summary of the data stratified by PI-RADS category and clinically significant biopsy status in Table 3.

**Table 3:** Summary by PI-RADS category and clinically significant biopsy status.

PI-RADS category	Clinically significant biopsy status	Patients (n)	Mean PSA (ng/mL)
2	No	1	12.83
	Yes	1	5.35
3	No	4	4.16
4	No	29	6.53
	Yes	14	6.84
5	No	3	8.62
	Yes	15	7.66

## DISCUSSION

Our findings support the utility of MVF analysis in evaluating PCa, similar to its applications in renal, hepatic, and cervical cancer. In MRI-targeted biopsies, MVF analysis appears to be a promising adjunctive tool because of its ability to identify areas of increased tumor vascularization, which may be associated with greater tumor aggressiveness (higher Gleason scores). The standardized grading of MVF in suspicious regions allowed for a more objective analysis of lesion vascularization and aided in selecting biopsy targets.

Major limitations of this pilot study include the modest sample size, lack of a separate control group, and the qualitative nature of MVF analysis. Future studies should incorporate quantitative MVF metrics and larger, controlled cohorts to confirm these preliminary results.

## CONCLUSION

The observed correlation between MVF and the Gleason score suggests that Doppler MVF analysis increases diagnostic accuracy by guiding biopsies toward potentially more aggressive regions of prostatic tumors. These are preliminary, hypothesis-generating findings and should therefore be validated in larger-scale studies.

## Data Availability

Data sets related to this article will be available upon request to the corresponding author.
